# Smart Compression Sock for Early Detection of Diabetic Foot Ulcers

**DOI:** 10.3390/s24216928

**Published:** 2024-10-29

**Authors:** Julia Billings, Julia Gee, Zinah Ghulam, Hussein A. Abdullah

**Affiliations:** 1 School of Engineering, University of Guelph, Guelph, ON N1G 2W1, Canada; jbilli01@uoguelph.ca (J.B.); geej@uoguelph.ca (J.G.); ghulamz@uoguelph.ca (Z.G.); 2 Scientific Research Centre, Australian University, West Mishref Mubarak Al-Abdullah Al-Jaber Area, Kuwait City 13015, Kuwait

**Keywords:** diabetic foot ulcers, diabetic neuropathy, plantar pressure, temperature, blood oxygen, medical device

## Abstract

The prevention of diabetic foot ulcers remains a critical challenge. This study evaluates a smart compression sock designed to address this issue by integrating temperature, plantar pressure, and blood oxygen sensors and monitoring data recorded by these sensors. The smart sock, developed with input from a certified Pedorthist, was tested on 20 healthy adult participants aged 16 to 53. It includes four temperature sensors and pressure sensors at common ulcer sites (first and fifth metatarsal heads, calcaneus, and hallux), and a blood oxygen sensor on the hallux. The sensors are monitored, and their transduced data are collected and stored through an app installed on a personal cell phone. The mobile app interface is user-friendly, providing intuitive navigation and easy access to the sensors’ data. Using repeated measures ANOVA and post hoc tests, we analyzed the impact of various physical activities on physiological changes in the foot. The device effectively detected significant variations in blood oxygen, temperature, and pressure across six activities. Statistical analyses revealed significant differences based on activity type and sensor location. These results highlight the smart sock’s sensitivity and accuracy, suggesting its potential to prevent diabetic foot ulcers. Further clinical trials are needed to evaluate its efficacy in a larger, more diverse population.

## 1. Introduction

### 1.1. Diabetic Foot Ulcers (DFUs)

Diabetes is rapidly emerging as a global epidemic, with over 425 million adults affected worldwide, projected to increase to 629 million by 2025 [1]. Diabetes mellitus is a chronic metabolic disorder characterized by the accumulation of glucose in the bloodstream, leading to hyperglycemia [2]. This condition often results in diabetic neuropathy, a prevalent complication that poses significant health risks for individuals with diabetes and places additional strain on the already overwhelmed healthcare system [2]. Diabetic neuropathy refers to nerve dysfunction resulting in decreased sensation and protective reflexes in the feet and legs [2]. This reduced sensitivity increases the risk of foot injuries and the development of foot ulcers, which are open wounds that are prone to infection, over time [3]. Due to diabetic neuropathy, individuals may overlook minor foot injuries such as calluses, cuts, blisters, burns, or ingrown toenails, all of which can progress to diabetic foot ulcers (DFUs) [4]. A foot ulcer is a break in the dermis or deeper levels of the skin, characterized by a loss of epithelium [5]. Moreover, inadequate treatment of these injuries due to lack of awareness can worsen the situation, leading to DFU formation. DFUs are a significant contributor to morbidity and nontraumatic lower limb amputations in those with diabetic neuropathy [6,7]. Approximately 33% of diabetic patients will develop a foot ulcer in their lives, with 17% ultimately requiring amputation [8]. Thus, DFUs not only affect a large portion of the diabetic population but also have fatal consequences. 

Undergoing an amputation is a life-altering event that can lead to persistent pain, phantom limb sensations, and profound emotional distress, including depression and anxiety [9]. These consequences profoundly affect the quality of life for diabetic individuals. A 2018 study revealed that the average annual cost of treating DFUs worldwide is approximately USD $8659 per patient, and with the global estimated increase in diabetes, this number is expected to grow [10]. In terms of health and safety, DFUs pose significant risks as they are prone to infection and even small traumas can have serious consequences if left untreated [4]. The early detection and appropriate treatment of DFUs can ultimately help prevent their occurrence in diabetic individuals [11]. Therefore, there is a pressing need for a solution that enables diabetic patients to detect foot ulcers during early onset.

### 1.2. Biomarkers Associated with DFUs 

Numerous biomarkers have been evaluated for their potential in detecting DFUs, including plantar pressure, temperature, blood oxygen, heart rate, and pH levels. By leveraging these biomarkers, the early detection of DFUs becomes feasible, potentially preventing the need for amputation.

Plantar pressure refers to the force distribution across the sole of the foot during daily activities [12]. Individuals with diabetic neuropathy commonly face elevated plantar pressures due to repeated and unnoticed pressure on weight-bearing surfaces of the foot [1]. A previous study confirmed that elevated plantar pressures on the foot cause ulceration in the presence of diabetic neuropathy [13]. Another study suggested that patients with diabetic neuropathy should aim to reduce their maximum plantar pressure by 30% to mitigate the risk of DFUs forming [14]. Offloading interventions, such as specialized footwear and insoles, have gained popularity for redistributing pressure away from the high-pressure areas of the foot, aiming to prevent DFUs [1,15]. In a study involving 47 patients, the average plantar pressure among those with diabetic neuropathy was found to be 205.3 +/− 118.6 kPa [16]. The average peak plantar pressure for adults ranges from 80 kPa to 600 kPa [17]. The high-pressure areas of the foot have been identified as the first and fifth metatarsal heads, the hallux, and the calcaneus [16]. This is because the central heel and the metatarsals bear the greatest loads, making them more prone to developing foot ulcers [18].

Skin temperature serves as another biomarker capable of early detection of DFUs [19]. Elevated skin temperatures of the foot may indicate inflammation, which anticipates the formation of a wound on the foot [20]. Research supports that an increase in foot temperature beyond a specific threshold will suggest inflammation of the foot [20]. A temperature difference of more than 4°F between a patient’s feet is a potential indicator of developing a foot ulcer [21].

Adequate oxygen supply is also crucial for effective wound healing and thus insufficient blood oxygen levels elevate the risk of infection and potential ulceration [22]. Normal blood oxygen levels are known to be between 95 and 100%; however, if oxygen levels at the wound site drop below this range, the risk of infection escalates [11,23]. Therefore, insufficient oxygen supply to the foot can impede wound healing following an injury, potentially leading to ulcer formation.

Research has also indicated that the maintenance of an acidic environment promotes effective wound healing by regulating and facilitating oxygen release [19]. For this reason, pH is also said to be a significant biomarker for evaluating DFUs. A study conducted in 2018 involving 50 patients, 34 of whom had diabetic neuropathy, suggested that a pH range of 7.6–7.8 could signify the presence of a DFU [20].

### 1.3. Existing Solutions for DFUs

Although a wide range of research has been conducted regarding DFUs, there is still a need for a better understanding of how to detect and prevent them. To prevent the formation of DFUs, diabetics are advised to inspect their feet daily and look for blisters, scratches, cuts, and ingrown toenails that could lead to the formation of an ulcer [24]. Diabetics are also advised to avoid walking barefoot and continually monitor their blood sugar levels as high blood sugar levels can lead to difficulties in the healing process [24]. Weight loss and the cessation of tobacco use are also encouraged, as excessive weight can place increased stress on the feet, and tobacco in the bloodstream has been found to reduce blood flow in the lower extremities [24]. Other existing methods of prevention include cold therapy, antibiotics, offloading devices, and surgery [5]. While these methods do exist, they are invasive, expensive, affect other body functions, and do not successfully detect the early signs of DFUs or provide constant monitoring of the wound [5]. Moreover, many of these prevention methods are reliant on the consistency of human behavior and with the predisposed presence of peripheral neuropathy in diabetics, any small change to the bottom of their foot can easily go unnoticed [24]. 

The current technological devices on the market to aid in the prevention of DFUs include instruments to record daily plantar temperature, socks that solely provide continuous temperature or pressure monitoring, alarm systems, activity monitoring devices, and smart insoles [6]. Siren Socks and Sensoria^®^ Socks, are the marketed socks that currently act as a wearable device to solely monitor an individual’s foot temperature and pressure, respectively [25,26]. Each of these current devices only uses one of the biomarkers found to be associated with DFUs and thus no comprehensive solution currently exists to continuously monitor the progression of DFUs by integrating data from multiple biomarkers. Thus, a device that can provide monitoring of several of the biomarkers associated with DFUs and is not reliant on the consistency of human behavior in the prevention process is needed. 

### 1.4. Proposed Design 

The proposed design’s main objective was to create an affordable and wearable medical device to allow continuous monitoring of early signs of DFUs. The design incorporates temperature, plantar pressure, and blood oxygen sensors and reinforced features including seamless toe and heel elements, a non-slip cuff, arch support, 30–40 mmHg graduated compression, and electronic components. A custom-made mobile app was developed using Arduino IDE and React Native programs to communicate and display the data from the sensors to the user continuously in real time. Bluetooth Low Energy (BLE) thus communicates between the microcontroller and app software. The sock underwent rigorous testing in a preliminary trial using a thorough testing procedure which was developed through literature reviews on previous clinical trials. 

## 2. Materials and Methods

### 2.1. Design of Device

The main focus of the design is to develop a wearable, comfortable, and usable device that can transduce a number of biomarkers, such as plantar pressure, temperature, and blood oxygen rate. The medical device design represented an intelligent graduated compression sock tailored for diabetic patients, featuring integrated temperature, plantar pressure, and oxygen saturation (SpO_2_) sensors to evaluate three biomarkers associated with foot ulcers. In this design, four textile temperature sensors were embedded into the fabric of the sock. Extensive research revealed that common ulcer locations on the foot include the first and fifth metatarsal heads, the calcaneus, and the hallux [27]. Consequently, the four temperature sensors were strategically embedded in these specific areas. Additionally, four plantar pressure sensors were incorporated into the fabric, in the same locations as the temperature sensors [18]. To introduce another biomarker for DFU detection, a blood oxygen sensor was attached to the big toe as this location receives the most consistent circulation of blood [27,28]. Overall, after consultation with a certified Pedorthist and extensive research, these locations were identified as optimal for sensor placement, aligning with the most common areas for foot ulcer development and blood oxygen readings [27]. The temperature, plantar pressure, and blood oxygen sensors were tested and calibrated per manufacturer data sheets before being mounted on the compression sock. This ensured accurate data reporting for temperature, pressure, and oxygen levels based on their operating principles.

**Figure 1:**
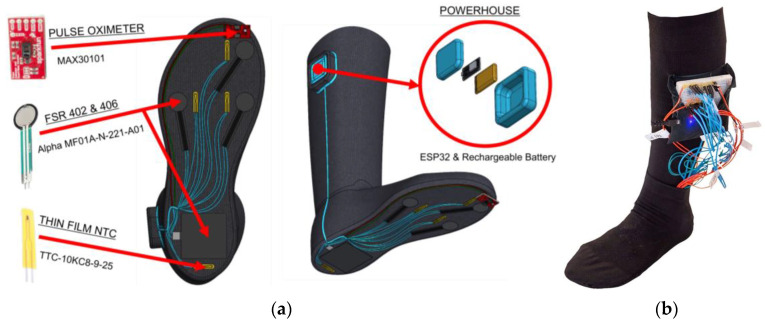
Final design of the device: (**a**) 3D overview and (**b**) Actual prototype built for data collection.

**Figure 2:**
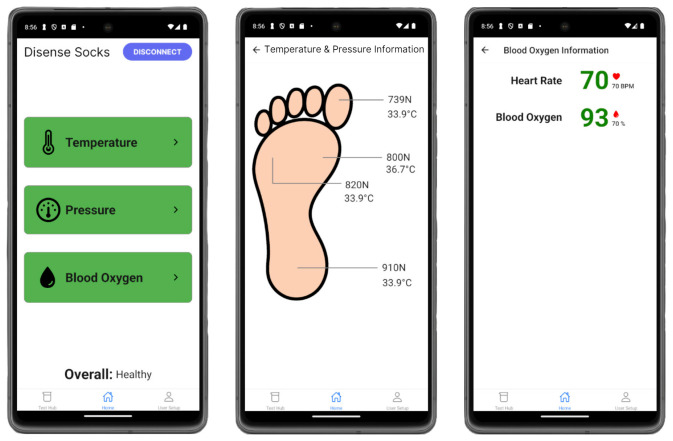
Mobile app interface developed for the sock to provide the user with data [29].

The temperature, plantar pressure, and SpO_2_ sensors are connected to an ESP32 microcontroller, and the battery is housed in a fabric-sewn pouch at the top of the sock (Figure 1). The battery is removable and can be recharged. The sensors transmit their readings to the microcontroller through an analog-to-digital converter (ADC). The microcontroller utilizes a Bluetooth Low Energy (BLE) module to transfer data to the mobile app. The app delivers real-time sensor data to the user, providing readings every 5 s on temperature and pressure at the four locations, heart rate, and the blood oxygen level of the foot. Sensor data are compared to literature-reviewed thresholds, and the app promptly displays to the user when these thresholds are surpassed, indicating the potential onset of an ulcer on the foot. The mobile app interface is user-friendly, providing intuitive navigation and easy access to sensor data. Each sensor is equipped with a button that remains green and switches to red when the threshold is exceeded as seen in Figure 2 [29]. The app integrates an “Overall” health feature that displays the status of the patient with three different outcomes: healthy, moderate risk, and high risk. The features within the custom-made app empower individuals with diabetes to share the data collected on the app with their healthcare provider for monitoring and early intervention to prevent DFUs.

Recognizing that user-friendly medical devices are in high demand, it was imperative to ensure that the smart compression sock design prioritized patient comfort [30]. This was achieved with materials such as anti-odor and moisture-wicking fabric, promoting dryness in the legs and feet. The compression sock used was made from 25% Supima cotton, 25% spandex, and 50% nylon. These materials were selected due to their high comfort level, breathability, moisture resistance, and ability to inhibit bacterial growth [31]. Additionally, they facilitate heat transfer between the skin and the smart compression sock through thermal conductivity, absorptivity, and air permeation. Numerous factors related to the thermal physiological properties of fabrics were considered, including fiber type, conductivity, moisture regain, structure, loop length, thickness, and porosity [32]. The sock’s material is crafted with a seamless knitted loop, providing additional comfort, and safeguarding high-friction areas of the foot [33]. Moreover, the smart compression sock employs 30–40 mmHg gradient compression to prevent swelling and improve blood circulation in the foot [33]. Therefore, the women’s large long black sock from OrthoMed Canada was chosen in the design of the device as it satisfied all these requirements [34]. The sock integrates temperature and plantar pressure sensors into its fabric, with blood oxygen sensors attached to the side of the big toe. These sensors are not visible on either side of the sock fabric, being covered with a thin layer of additional fabric commonly used in manufacturing processes [35]. To enhance sensor readability, small mesh viewports are strategically placed where the blood oxygen sensors are located [36]. Yarns are meticulously sewn into the outer fabric of the sock, ensuring that comfort is not compromised [35]. This design also incorporates small mesh viewports at the locations of the blood oxygen sensors, further optimizing sensor readability accuracy.

#### 2.1.1. Temperature Sensors

The smart compression sock has four thin-film, flexible temperature sensors sewn into the textile fabric that is situated alongside the 1st and 5th metatarsal heads, hallux, and the calcaneus [35]. The TTC-10KC8-9-25 Thermistor is the temperature sensor selected for the design [37]. The temperature sensors were calibrated to ensure various temperature output resistances within the resistance tolerances and could be read by the sensors [37]. A voltage divider circuit was used for the thermistors to convert the resistance of the thermistor to a voltage value for the ADC to read. The voltage divider found the output voltage [17]. The resolution of the ADC in the microcontroller converts the voltage read by the ADC to an analog voltage. The resistance value of the thermistor was calculated using the voltage divider formula. To acquire an accurate temperature value, the thermistor resistance and the lookup table provided by the datasheet were used, and then linear interpolation was conducted on the resistance value less than and greater than the thermistor resistance. The smart compression sock will notify patients if the temperature difference between the patient’s two feet is greater than 4°F, as this difference signifies that the patient has the potential to develop a foot ulcer [21].

#### 2.1.2. Plantar Pressure Sensors 

Four force sensitive resistor (FSRs) sensors were sewn into the textile fabric of the smart compression sock that is situated alongside the 1st and 5th metatarsal heads, hallux, and calcaneus to measure the plantar pressure. FSRs consist of 2 outer substrate layers, an inner conductive film, and a spacer which changes in resistance when a force is administered [38]. Deformation of the conductive film occurs when a high force is applied to the FSR, and the substrate layers act to decrease the resistance [38]. The force sensors collect real-time data of various areas of the foot where force is exerted and convert the force into a pressure value [39]. The smart compression sock alerts patients when plantar pressure values on the area of the foot exceed the set 143 kPa threshold. This threshold was chosen because the average plantar pressure for a person with diabetic neuropathy is 250 kPa, and it is suggested that those with this condition reduce their pressure by 30% to 175 kPa [16]. Based on the recommendation from the certified Pedorthist, it was decided to provide a safety buffer, so the threshold of 143 kPa was selected beyond the 175 kPa target.

The active sensing area of the FSR-402 by Interlink Electronics has also been found to be 126.68 squared millimeters due to a radius of 6.35 mm [17]. Knowing 1 pascal is equivalent to 1 newton per meter squared of force, the average peak adult plantar force ranges can be calculated. The FSR sensors output a resistance value based on the amount of force applied to the sensor. When no force is applied, the resistance is equivalent to infinity, and as more force is applied, the resistance (R) approaches zero. [13,15]. A voltage divider circuit will be needed for each FSR to convert the resistance output to a voltage value as the microcontroller’s ADC can only measure voltage. The supply voltage is equal to 3.3 V, and the R_o_ value selected for the force sensor is 1 kΩ as this resistance is known to provide the optimal response in a voltage divider circuit based on the range of force output by a human foot [17]. The digital voltage read by the ADC will be converted back into an analog voltage value. The voltage value will then be converted back to resistance after it is read by the microcontroller by rearranging the voltage divider formula for FSR. A calibration process was conducted using the voltage divider circuit to obtain a resistance-to-force correlation. Using the data from the calibration process, a resistance to force function was calculated.

#### 2.1.3. Blood Oxygen Sensor

One blood oxygen sensor is placed on the plantar aspect of the interphalangeal joint. The sensor emits two lights through the skin and measures the amount of reflected light using the photodetector [36]. Light emitted through the skin can be used to compare the amount of red light and infrared light (IR) absorbed by the blood [36]. A pulse oximeter is a device that measures oxygen saturation, known as SpO_2_ [36]. The Di-Sense smart compression sock will measure blood oxygen levels on the foot by embedding a pulse oximeter. The Maxim Integrated MAX30101 sensor is the blood sensor selected as it is a low-cost sensor that outputs the blood oxygen levels of the blood as a percentage and is widely used in several wearable devices [40]. The MAX30101 consists of 2 internal LEDs, photodetectors, optical elements, and a low-noise analog signal process to detect blood oxygen levels [40]. Its low noise feature meets the criteria to ensure minimal noise during operation. The output data from the MAX30101 can be stored in 32-deep First in First Out (FIFO) in the integrated circuit [40]. The smart compression sock will notify participants if blood oxygen in the foot reaches levels below 94% since healthy blood oxygen levels range between 95 and 100% [41].

#### 2.1.4. Microcontroller and Communication Protocol

The ESP32-WROOM-32 is the microcontroller used in the design [42]. The ESP32 development board contains the D0WDQ6 chip, which offers dual cores, each running at 240 MHz, low power consumption with multiple sleep modes, Bluetooth 4.2, a 12-bit analog-to-digital converter (ADC), and 18 pins that use the ADC [42]. This ESP32 board was selected as the 12-bit ADC, and the number of ADC pins allowed the connection of the eight analog sensors (FSRs and thermistors) without needing a multiplexer (MUX). The ESP32 has a chip and pins that support digital sensors (Serial Data (SDA) and Serial Clock (SCL)), which allows for the support of the blood oxygen sensor (MAX30101). The chip has a deep sleep mode, uses a minimal amount of power, has Bluetooth 4.2, and supports Bluetooth Low Energy (BLE), allowing for energy efficient communication between the device and the mobile app. The BLE is within 10 m–40 m, which is adequate for the device’s purposes [17].

#### 2.1.5. Power Supply and Data Acquisition System 

The smart compression sock’s Powerhouse and dock maintain battery life and manage sensor outputs. The power supply will contain a 3.7 V lithium polymer (Li-Po) rechargeable battery [17]. Li-Po batteries produce a current large enough for the ESP32′s BLE Tx and Rx outputs, and an excellent charge efficiency [17]. The battery selected has a current capacity of 600 mAh and circuitry to protect against overcharging and lower the discharge rate.

### 2.2. Flow Diagram

**Figure 3:**
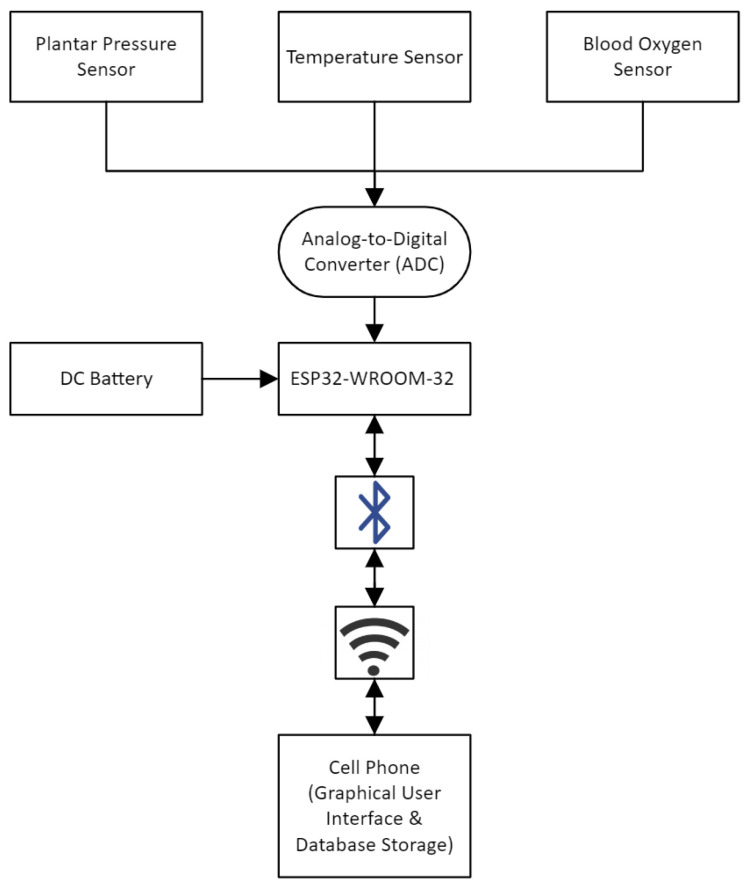
Flow diagram of the device.

Figure 3 shows the smart compression sock’s output process and integration with the ESP32 microcontroller and cell phone for the plantar pressure, temperature, and blood oxygen sensors.

### 2.3. Experimental Goals

The first experimental goal of this device was to create a functional smart compression sock capable of detecting changes in temperature, plantar pressure, and blood oxygen levels, in the correct locations on the foot. Ensuring the ability of each sensor to detect values at their specific locations proves that if changes from the healthy baseline occur, this will be detected in the correct location where that sensor is located on the foot. Confirming the ability of each sensor to read and output values at their specific spot on the foot will allow this area to be raised as a cause for concern and addressed accordingly. Secondly, the device intends to evaluate the sensitivity of each sensor and their individual abilities to detect small changes in temperature, blood oxygen, and plantar pressure in their area of the foot. A higher sensitivity of the sensors will show that even the smallest deviation from the healthy baseline could be cause for concern. The device can be considered successful if each sensor functions in its specific area of the foot and is sensitive to small deviations from the healthy baseline. Giving participants the ability to locate the problem in a timely matter will ultimately provide users with ease and allow them to contact their healthcare provider to take appropriate action.

### 2.4. Methods

#### Procedure

The study included 20 participants, comprising 16 females and 4 males, aged between 16 and 53 years. The participants were selected to represent various body types and shoe sizes. Each participant’s age, weight, height, and shoe size were recorded. 

Certain inclusion criteria had to be met to be eligible as a participant in this study. The first of these criteria was the ability to provide valid informed consent. The second criterion was the ability to communicate well in English. The third inclusion criterion was to be capable of undergoing daily human movements where temperature, blood oxygen, and plantar pressure changes occur in their lower extremities. The final criterion was for the participant to have an appropriate foot size to fit into the device, ranging from men’s US size 7.5–12 and women’s US 9.5–14 [34].

Participation in the study was voluntary. A participant package was provided, including an information letter explaining the testing procedure, a consent form, and an exit survey. If all inclusion criteria were met and the participant signed the consent form and agreed to the procedure outlined, they were allotted a spot to conduct testing with the device. The rights of the research participants were explained to each participant, and before beginning any testing, each participant was asked if they would like to participate in the study. Each component of the device and its function were explained. A cleared smartphone, specifically intended for use in this project, was set up with the software and application needed to connect all the sensors and monitoring. Data were collected on this smartphone through Bluetooth and transferred through Wi-Fi to a locally stored private OneDrive within the University of Guelph for testing.

The participant was provided with the sock, instructions on how to put the smart sock on, and the mobile phone with the app needed for monitoring and data collection. The testing phase then began. Participants were instructed to wear socks for at least 1.5 h for testing. The participants put the sock on their right foot following observation and assistance by the team. All sensors were turned on and checked for function and reading. If one sensor was not working, it was fixed or replaced, and then the participant put the sock back on their right foot. The participant was shown how to connect the device via Bluetooth for the duration of testing.

The participants entered their weight, height, and shoe size into the mobile app, which then created a unique ID number. The participants then went about daily activities for the next 1.5 h. The participants performed each of the following six activities, including jogging, lying down, sitting with feet on the ground, sitting with feet up, standing, and walking for at least 15 min for each of these activities. The participants recorded their activity by selecting it in the app’s graphical user interface. This selection ensures that the collected sensor data and associated timestamps are recorded in the database. Blood oxygen levels, heart rate, temperature, and plantar pressure were collected through the device’s different sensors during testing.

After an average of 1.5 h elapsed, the participant returned the sock and completed the exit survey to provide feedback on design comfort, ease of use, and functionality. All data were then transferred from the smartphone to a private OneDrive. The research study and trial have been reviewed and approved by the University of Guelph Research Ethics Board (REB# 24-01-012) for compliance with federal guidelines for research involving human participants.

## 3. Results

### 3.1. Participants’ General Characteristics

The trial involved 20 participants (4 males, 16 females), and their anthropometric characteristics are listed in Table 1.

This study provides a comprehensive statistical analysis of the effects of diverse physical activities on key physiological parameters—namely oxygen saturation, foot temperature, and pressure—across multiple sensor locations on the foot. Through the application of repeated measures ANOVA and subsequent post hoc tests, we examined how different activities influence these parameters. Our findings reveal significant alterations in oxygen levels corresponding to varying levels of physical exertion, with marked differences observed across activity types. Additionally, temperature and pressure readings at four distinct foot sensor locations (1st and 5th metatarsal heads (M1 and M5), Calcaneus, and Hallux) exhibited notable interactions with the type of activity performed. These results emphasize the complex interplay between physical activity and physiological responses at different foot locations. 

### 3.2. Temperature Results Analysis

Temperature data were analyzed from twenty participants performing six different activities at four distinct foot locations. Table 2 shows the mean temperature averaged across all locations and participants for each activity. 

Table 3 illustrates the average temperature by sensor foot location averaged across all participants.

**Figure 4:**
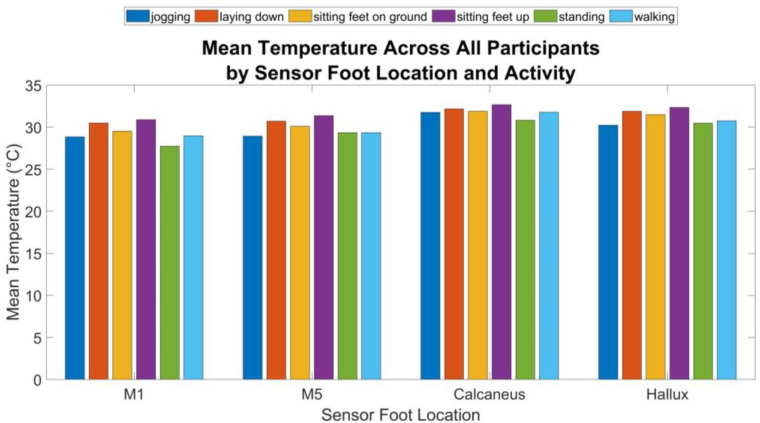
Mean temperatures across all participants by sensor foot location for Activity.

Figure 4 shows the mean temperature across all participants by sensor foot location for each activity.

#### 3.2.1. Normality Assessment for Temperature 

Normality was assessed for each activity and foot location combination using the Shapiro–Wilk test. Results indicated that most groups met the normality assumption, except for two groups: jogging at M1 and standing at M1. These groups exhibited *p*-values below 0.05, suggesting deviations from normality. Despite these deviations, no data transformations were performed due to the overall distribution of the data. 

#### 3.2.2. Repeated Measures ANOVA for Temperature

The repeated measures ANOVA was used to evaluate the effects of activity, location, and their interaction on temperature readings. Mauchly’s Test for Sphericity was used to test whether the assumption of sphericity is met in a repeated measures ANOVA. The test indicated violations (*p*-value was less than 0.05) for all effects (activity, location, activity x location), necessitating the use of Greenhouse-Geisser (GG) and Huynh-Feldt (HF) corrections. The analysis revealed significant effects as can be seen in Table 4. 

The results indicated that there is a significant effect among activity, location, and the interaction between activity and location. 

#### 3.2.3. ANOVA Results by Foot Location

Table 5 displays the results for the separate ANOVAs for each foot location.

#### 3.2.4. Post Hoc Comparisons

**Figure 5:**
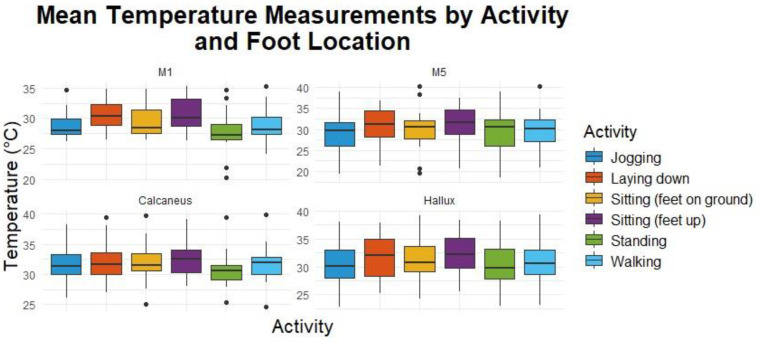
Foot temperature boxplot with pairwise comparisons.

Pairwise comparisons using Bonferroni-adjusted *t*-tests revealed significant differences between several activities at different foot locations. Effect sizes for these comparisons were calculated to assess the magnitude of the differences. Figure 5 presents the mean temperature for each foot location by activity state across all the participants.

### 3.3. Pressure Results Analysis

Data collected by the pressure sensors were analyzed from twenty participants performing six different activities at four distinct foot locations. Table 6 shows the mean force averaging across all locations and participants for each activity before and after normalization. Normalization was achieved by dividing the mean force exerted from the participant’s foot by their body weight. All the normalized mean forces were then averaged across all the participants to produce the results in Table 6 and Table 7. 

Table 7 illustrates the average force acquired by the pressure sensors while performing various activities before and after normalization. 

**Figure 6:**
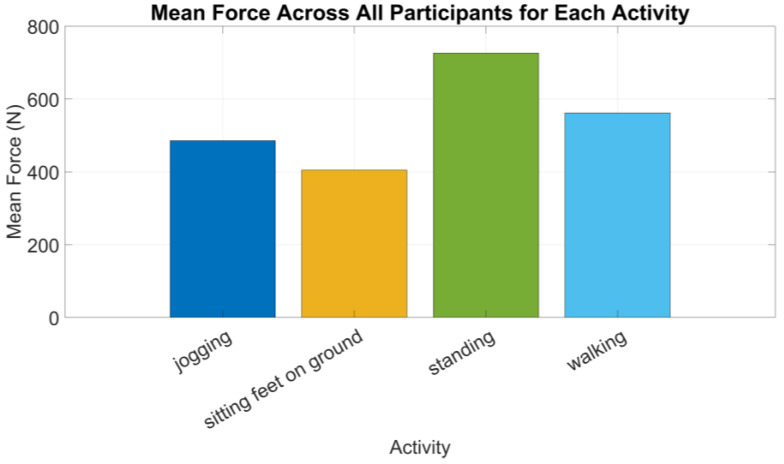
Mean force across all participants for each activity.

Figure 6 shows the force by sensor foot location for all activities for all participants. The two activities—laying down and sitting with feet up—were excluded from Figure 6 because the sensors are not in direct contact with the ground and therefore the values were 0 N (as could be seen in Table 7).

#### 3.3.1. Shapiro–Wilk Test Results

Shapiro–Wilk tests were conducted to assess the normality of force-sensitive resistor (FSR) data across different activities and foot sensor locations. Results indicated that several groups deviated significantly from normality, as evidenced by *p*-values less than 0.05. 

#### 3.3.2. Kruskal–Wallis Rank Sum Results

Due to deviations from normality, the Kruskal–Wallis rank sum test was employed to assess differences in FSR measurements across various activities and sensor locations. The Kruskal–Wallis test for activities yielded a chi-squared value of 346.68 with 5 degrees of freedom and a *p*-value < 2.2 × 10^−16^, indicating significant differences in FSR measurements between different activities (Table 8). This finding is consistent with previous studies demonstrating that physical activities significantly affect pressure measurements due to variations in foot mechanics and load distribution [43].

#### 3.3.3. Kruskal–Wallis Rank Sum Results for Activities

To determine which specific activities and locations differ significantly from each other, post hoc pairwise comparisons were performed with Bonferroni adjustment, as evident in Table 9.

Significant differences were observed between jogging and all other activities, with *p*-values less than 2.0 × 10^−16^. Laying down differed significantly from jogging, sitting with feet on the ground, standing, and walking. However, the comparison with sitting with feet up was not significant (*p* = 0.44370). Sitting with feet on the ground showed significant differences compared to all other activities, while sitting with feet up differed significantly from all other activities, with adjusted *p*-values ranging from 3.0 × 10^−7^ to 2.0 × 10^−16^. Standing showed significant differences between jogging, lying down, sitting with feet on the ground, and sitting with feet up, but not from walking (*p* = 1.000).

#### 3.3.4. Pairwise Comparisons of Activities 

**Figure 7:**
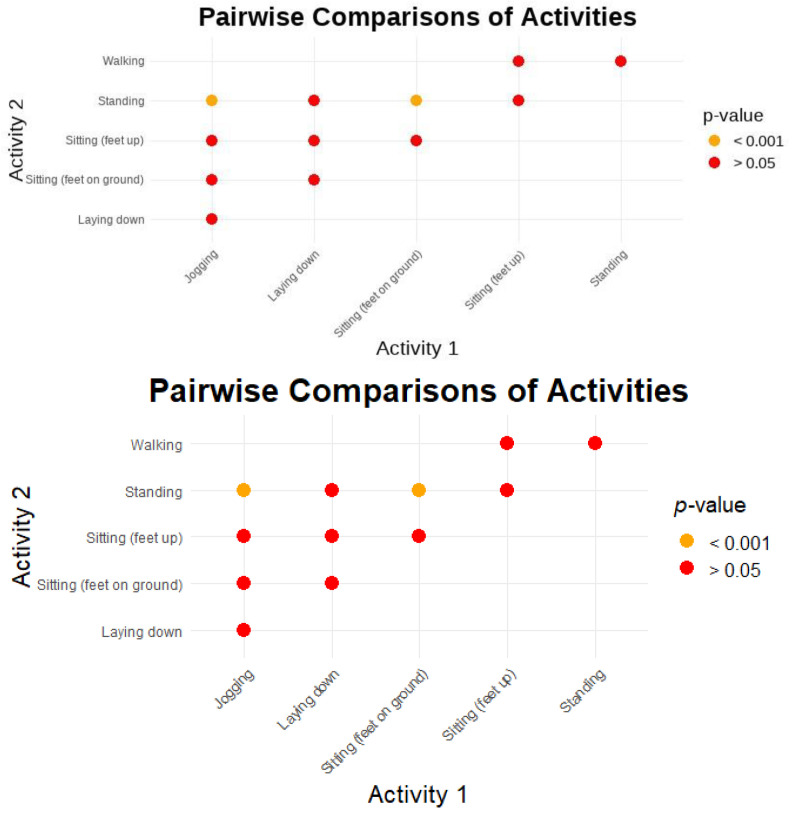
Pairwise comparisons of activities.

Pairwise comparisons of one activity with another are represented in Figure 7 by comparing the significance of each effect (i.e., *p* < 0.001 indicates no significance).

#### 3.3.5. Kruskal–Wallis Rank Sum Results for Locations

For sensor locations, the Kruskal–Wallis test showed a chi-squared value of 40.966 with 3 degrees of freedom and a *p*-value of 6.6 × 10^−9^, reflecting significant differences in FSR measurements across different sensor locations (Table 8). This result aligns with the literature, suggesting that sensor placement on different foot areas yields varying pressure readings due to anatomical and functional differences [44]. 

Post hoc comparisons for sensor locations showed significant differences, which are evident in Table 10.

Significant differences were observed between M1 and Calcaneus, and between M5 and Calcaneus, reflecting the impact of sensor placement on pressure readings. This finding is in line with previous studies indicating variations in pressure measurements exist across different foot locations [45].

The force distribution varies significantly by both activity and sensor location. Walking and jogging generally produce the highest force values. The calcaneus sensor records the highest range of force measurements, particularly during walking, indicating a higher load on this area of the foot during such activities. Sitting and laying down activities produce the lowest force values across all sensor locations, with minimal variation, as expected due to the reduced load on the feet during these activities.

#### 3.3.6. Pairwise Comparisons of Locations 

**Figure 8:**
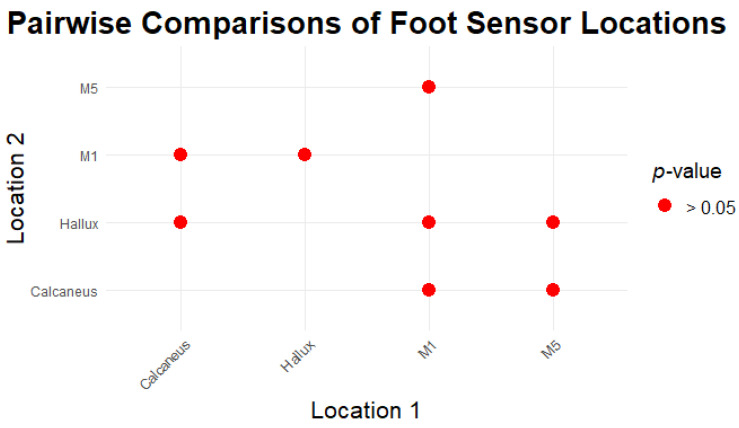
Pairwise comparisons of foot sensor locations.

Figure 8 represents pairwise comparisons of one sensor location with another by comparing the significance of each effect (i.e., *p* > 0.05 indicates significance). 

#### 3.3.7. Pairwise Comparison for Pressure by Activity and Location

**Figure 9:**
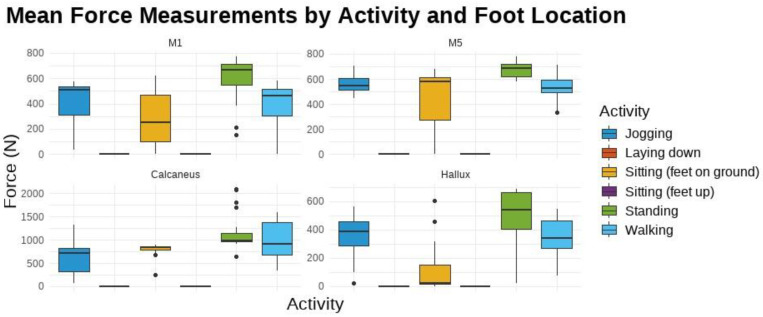
Mean FSR by Activity State Across All Participants.

Figure 9 presents the mean FSR for each foot location by activity state across all the participants.

### 3.4. Oxygen Results Analysis

**Figure 10:**
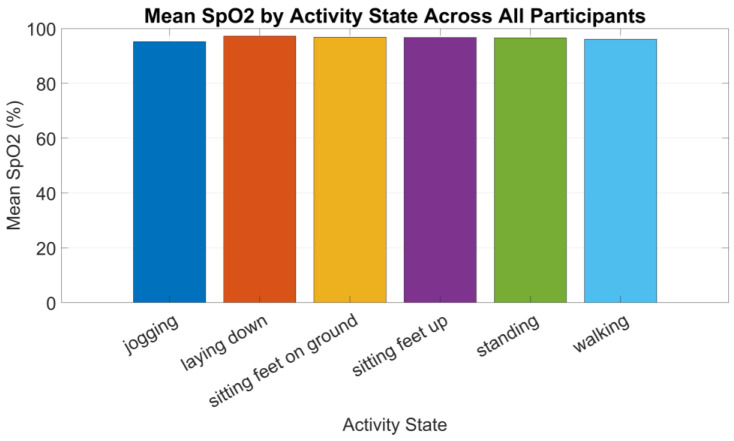
Mean SpO₂ by activity state across all participants.

Figure 10 shows the data collected by the oxygen sensor we analyzed and the average SpO₂ by activity state across all participants.

#### 3.4.1. Normality Assessment for Oxygen 

The Shapiro–Wilk normality test was conducted to assess the normality of oxygen levels during each activity. All *p*-values were above the 0.05 threshold, suggesting that the oxygen level data were approximately normally distributed across different activities, as evident in Table 11.

#### 3.4.2. Repeated Measures ANOVA Oxygen

A repeated measures ANOVA was performed to determine if there were significant differences in oxygen levels across various activities. The analysis revealed a significant main effect of activity on oxygen levels, with an F-value of 4.528 (df = 5, 95) and *p*-value of 0.000956. The generalized χ^2^ (ges) for this effect was 0.1498, indicating a significant activity effect on oxygen levels.

#### 3.4.3. Sphericity Testing

Mauchly’s test for sphericity indicated that the assumption of sphericity was violated (W = 0.016, *p* < 0.001). Table 12 shows results after applying sphericity corrections using Greenhouse–Geisser (GGe) and Huynh–Feldt (HFe) methods. 

After applying the corrections, the *p*-values were still significant, indicating that the results remain significant even after accounting for the violation of sphericity. 

#### 3.4.4. Post Hoc Pairwise Comparisons

To further investigate the differences between activities, pairwise *t*-tests with Bonferroni correction were performed. However, none of the pairwise comparisons reached statistical significance, suggesting that while overall activity had a significant effect on oxygen levels, the specific pairwise differences were not significant after correction. 

## 4. Discussion

### 4.1. Temperature Results

The repeated measures ANOVA indicated significant temperature variations across different activities and foot locations, with a notable interaction effect between these factors. Previous studies have shown that physical activities lead to significant changes in foot temperature, with variations depending on the type of activity and the specific location of the foot [46].

The significant effect of activity on temperature aligns with known physiological changes induced by activities like jogging and standing, which are associated with increased metabolic rates and blood flow, leading to localized temperature changes [47]. A study on foot temperature variation in diabetic patients also supports the impact of physical activity on foot temperature, highlighting that activities such as jogging can cause higher temperature elevations due to increased blood flow and metabolic activity [48].

The significant differences in the temperature data collected by the proposed device across foot locations highlight the anatomical and physiological variations in temperature consistent with studies showing that different foot regions experience varying temperature changes due to differences in tissue composition and blood supply [49]. For example, the hallux, metatarsals, and heel area show higher temperatures relative to other locations on the foot, which aligns with other studies that show these locations tend to have the highest DFU rates [50].

The factorial ANOVA results suggest that both activity and foot location significantly influence temperature readings, with an interaction effect indicating that temperature differences between activities are not uniform across foot locations. This is particularly relevant for understanding the development of DFUs, as certain activities may cause higher temperature changes at specific foot locations, potentially exacerbating risk factors for DFUs. The research supports the approach that monitoring foot temperature could be a valuable tool in preventing DFUs, particularly in diabetic patients who are at risk of complications [51]. The research supports the approach that monitoring foot temperature could be a valuable tool in preventing DFUs, particularly in diabetic patients who are at risk of complications [51].

While these findings are valuable, it is essential to acknowledge the study’s limitations, such as deviations from normality in some groups (2/24 groups), which could affect the robustness of the results. However, the overall patterns remain consistent. Future studies could benefit from larger sample sizes and additional foot locations to further explore the effects observed and strengthen the understanding of how activity and foot location interact to influence temperature.

### 4.2. Pressure Results

Jogging shows significant differences in FSR when compared to most other activities except walking and sitting with feet on the ground. This distinct effect is due to the high-impact nature of jogging, which generates substantial pressure, especially under the metatarsals and hallux, leading to elevated FSR values. The research findings support that jogging produces higher peak pressures than walking or sitting because of its dynamic forces [52].

Non-weight-bearing activities like laying down and sitting with feet up consistently show lower pressure readings compared to other activities, due to minimal weight-bearing and reduced pressure across the foot. This aligns with studies in the literature indicating that positions like laying down significantly reduce foot pressure compared to activities that involve more dynamic shifts in weight distribution, such as standing or walking [53].

Standing also shows significant differences from walking, likely due to its static nature, which results in different pressure distributions. Static postures like standing can lead to prolonged pressure on specific foot regions, increasing the risk of pressure-related injuries [54].

Significant differences in FSR readings were also observed between sensor placements, particularly between M1 (metatarsal 1) and the calcaneus, and between M5 and the calcaneus. This suggests that sensor location greatly influences FSR readings, as different foot regions experience distinct pressure patterns during various activities. The metatarsal regions, involved in weight-bearing and propulsion, generally experience higher pressures compared to the calcaneus [55]. 

The pressure results analysis indicates that foot sensor readings are sensitive to both the type of activity and the specific foot location being measured. The variation across locations is likely due to anatomical and biomechanical differences in how pressure is distributed during different activities. For instance, pressure dynamics in the hallux differ from those in the calcaneus during activities like walking vs. standing. The repetitive, high-impact nature of jogging generates substantial pressure and stress across the foot, particularly in areas like the metatarsals and the hallux. These dynamic forces result in elevated pressure values, differentiating jogging from lower-impact activities like sitting. The pairwise comparison analysis of the pressure results further illustrates this finding. Certain activities consistently produce different FSR readings. Jogging often showed significant differences when compared to other activities, reinforcing the idea that it has a unique impact on pressure values across multiple foot locations. 

These results have implications for foot health, particularly in designing interventions or devices for monitoring foot pressure. Activities that produce significant differences in pressure readings could inform strategies to prevent conditions such as DFUs or pressure sores. Identifying which activities have the most pronounced effects on different foot locations can help in creating targeted strategies to mitigate adverse outcomes, such as custom footwear or pressure-relief insoles designed to reduce high-pressure areas. 

However, the study was limited to specific activities and locations. Future research could expand on this by including a broader range of activities, examining additional foot locations, and integrating other variables like footwear type, shear, moisture, surface hardness, or foot morphology to better understand foot pressure dynamics.

### 4.3. Oxygen Results

The oxygen results demonstrate a significant overall effect of physical activity on oxygen levels, particularly highlighted by the repeated measures ANOVA. However, the lack of significant pairwise differences after correction suggests that the effect might be subtle. The violation of sphericity and the borderline normality in the walking activity point to potential data variability, which could be addressed by increasing the sample size or refining the measurement methods.

The post hoc pairwise comparisons did not reveal any significant differences between specific activities after Bonferroni correction, suggesting that while there might be a general trend, the specific activities do not significantly differ from each other in their impact on oxygen levels. This is consistent with previous research findings indicating that physical activity can influence peripheral oxygen levels, but the degree of change may depend on the intensity and duration of the activity [56]. 

Further studies, possibly involving a larger and more diverse sample, are necessary to confirm these preliminary findings and explore the clinical implications for populations at risk of DFUs.

## 5. Conclusions 

This study assessed the effectiveness of a smart compression sock integrated with temperature, plantar pressure, and SpO_2_ sensors in detecting deviations from a healthy baseline that could indicate potential foot ulcers. The temperature sensors embedded at the first and fifth metatarsal heads, hallux, and calcaneus reliably detected localized heat changes, crucial for early ulcer detection. Similarly, the plantar pressure sensors accurately recorded forces exerted on different foot regions. The data showed significant differences in pressure measurements across various activities and sensor locations, validating the utility of FSR sensors in detecting pressure anomalies that could lead to ulceration. This confirms their alignment with established guidelines for diabetic foot care. 

The SpO_2_ sensor positioned on the hallux successfully monitored blood oxygen levels, a novel parameter in ulcer prevention. The device detected variations in oxygen saturation, contributing to a more comprehensive approach to ulcer risk assessment, as hypoxia in foot tissues can indicate compromised blood flow and increased ulcer risk. 

By combining temperature, pressure, and oxygen monitoring, the device addresses critical risk factors associated with diabetic foot ulcers and provides a foundation for future advancements in diabetic foot care technology. The integration of multiple sensors into a single device offers real-time alerts and a user-friendly mobile app interface, enhancing proactive management of foot health. Future research should investigate the long-term effectiveness of this smart compression sock across diverse populations and conditions. Clinical trials are needed to confirm its efficacy in preventing foot ulcers across different patient demographics and activity levels. Further optimization of sensor sensitivity and alert thresholds could also enhance the device’s performance and user experience. 

In summary, this study supports the potential of a smart compression sock with integrated sensors as a promising tool for preventing ulcers in diabetic patients. 

## Figures and Tables

**Table 1:** General characteristics of participants (n = 20).

Weight Group	Age (y)	Height (cm)	Weight (kg)	BMI (kg/m^2^)	Gender
Normal	22.29 ± 4.51	165.75 ± 6.16	57.47 ± 6.48	20.91 ± 1.71	M (2), F (15)
Overweight	21.50 ± 0.71	171.00 ± 5.00	70.50 ± 6.36	27.40 ± 1.45	M (1), F(1)
Obese	23.00 ± 0.00	178.00 ± 0.00	100.00 ± 0.00	31.60 ± 0.00	M (1), F (0)

**Table 2:** Mean temperatures averaged across all locations and participants for each activity.

Location	Mean Temperature (°C)
M1	29.64
M5	30.78
Calcaneus	31.86
Hallux	31.61

**Table 3:** Mean temperatures averaged across all Locations and participants for each activity.

Activity	Mean Temperature (°C)
Jogging	29.94
Laying Down	31.31
Sitting with Feet on the Ground	30.75
Sitting with Feet Up	31.82
Standing	29.60
Walking	30.21

**Table 4:** ANOVA results for the effects of activity, location, and their Interaction.

Effect	F(df1, df2)	*p*-Value	Ges
Activity	F(5, 95) = 8.99	*p* < 0.001	0.045
Location	F(3, 57) = 5.38	*p* = 0.003	0.068
Activity × Location	F(15, 285) = 2.96	*p* < 0.001	0.006

**Table 5:** ANOVA results for the effect of activity at different foot locations.

Location	Effect of Activity	F(df1, df2)	*p*-Value	Ges
M1	Significant	F(5, 95) = 10.64	*p* < 0.001	0.145
M5	Significant	F(5, 95) = 7.860	*p* < 0.001	0.033
Calcaneus	Marginally Significant	F(5, 95) = 2.750	*p* = 0.023	0.035
Hallux	Significant	F(5, 95) = 8.330	*p* < 0.001	0.039
M1	Significant	F(5, 95) = 10.64	*p* < 0.001	0.145
M5	Significant	F(5, 95) = 7.860	*p* < 0.001	0.033
Calcaneus	Marginally Significant	F(5, 95) = 2.750	*p* = 0.023	0.035
Hallux	Significant	F(5, 95) = 8.330	*p* < 0.001	0.039
M1	Significant	F(5, 95) = 10.64	*p* < 0.001	0.145
M5	Significant	F(5, 95) = 7.860	*p* < 0.001	0.033
Calcaneus	Marginally Significant	F(5, 95) = 2.750	*p* = 0.023	0.035

**Table 6:** Mean force averaged across all locations and participants for each activity before and after normalization.

Location	Mean Force (N)	Normalized Mean Force (N/kg)
M1	270.34	4.552
M5	362.11	5.885
Calcaneus	583.36	9.430
Hallux	218.64	3.594

**Table 7:** Mean force averaged across all locations and participants for each activity before and after normalization.

Activity	Mean Force (N)	Normalized Mean Force (N/kg)
Jogging	485.6	7.430
Laying Down	0.000	0.000
Sitting with Feet on the Ground	404.8	5.939
Sitting with Feet Up	0.000	0.000
Standing	726.2	12.35
Walking	562.0	9.969

**Table 8:** Kruskal–Wallis test results.

Factor	Chi-Squared	df	*p*-Value
Activity	346.68	5	<2.2 × 10^−16^
Location	40.966	3	6.6 × 10^−9^

**Table 9:** Post hoc pairwise comparisons of FSR measurements across activities.

Comparison	*p*-Value
Jogging vs. Laying Down	<2.0 × 10^−16^
Jogging vs. Sitting with Feet Up	<2.0 × 10^−16^
Jogging vs. Standing	3.0 × 10^−7^
Sitting with Feet on the Ground vs. Laying Down	<2.0 × 10^−16^
Sitting with Feet on the Ground vs. Sitting with Feet Up	<2.0 × 10^−16^
Sitting with Feet on the Ground vs. Standing	1.8 × 10^−6^
Sitting with Feet Up vs. Walking	<2.0 × 10^−16^
Standing vs. Walking	1.2 × 10^−4^
Standing vs. Sitting with Feet Up	<2.0 × 10^−16^
Walking vs. Laying down	<2.0 × 10^−16^

**Table 10:** Post hoc pairwise comparisons of FSR measurements across locations.

Comparison	*p*-Value
Calcaneus vs. M1	3.1 × 10^−5^
Calcaneus vs. M5	2.9 × 10^−4^
Hallux vs. M5	5.9 × 10^−4^
Hallux vs. Calcaneus	4.4 × 10^−6^
M5 vs. M1	9.0 × 10^−2^
Hallux vs. M1	7.9 × 10^−1^

**Table 11:** Shapiro–Wilk normality test results for oxygen levels across activities.

Activity	W Statistic	*p*-value	Normality
Jogging	0.9267	0.1334	Normal
Laying down	0.9684	0.7211	Normal
Sitting (feet on the ground)	0.9316	0.1660	Normal
Sitting (feet up)	0.9642	0.6298	Normal
Standing	0.9474	0.3298	Normal
Walking	0.9066	0.0550	Borderline normal

**Table 12:** Sphericity corrections for repeated measures ANOVA on oxygen levels across activities.

Correction	Corrected *p*-Value
GGe	0.0195
HFe	0.0160

## Data Availability

The data supporting this study’s findings are available on request.
